# Electro-Responsive Conductive Blended Hydrogel Patch

**DOI:** 10.3390/polym15122608

**Published:** 2023-06-08

**Authors:** Jang Ho Ha, Jae Hyun Lim, Jong Min Lee, Bong Geun Chung

**Affiliations:** 1 Department of Mechanical Engineering, Sogang University, Seoul 04107, Republic of Korea; 2 Research Center, Sogang University, Seoul 04107, Republic of Korea; 3 Division of Chemical Industry, Yeungnam University College, Daegu 42415, Republic of Korea; 4 Institute of Smart Biosensor, Sogang University, Seoul 04107, Republic of Korea

**Keywords:** electro-responsive blended hydrogel, alginate, gelatin methacrylate, silver nanowire, drug release

## Abstract

The proposed electro-responsive hydrogel has great benefit for transdermal drug delivery system (TDDS) applications. To improve the physical or chemical properties of hydrogels, a number of researchers have previously studied the mixing efficiencies of the blended hydrogels. However, few studies have focused on improving the electrical conductivity and drug delivery of the hydrogels. We developed a conductive blended hydrogel by mixing alginate with gelatin methacrylate (GelMA) and silver nanowire (AgNW). We demonstrated that and the tensile strength of blended hydrogels were increased by a factor of 1.8 by blending GelMA and the electrical conductivity was enhanced by a factor of 18 by the addition of AgNW. Furthermore, the GelMA-alginate-AgNW (Gel-Alg-AgNW) blended hydrogel patch enabled on-off controllable drug release, indicating 57% doxorubicin release in response to electrical stimulation (ES) application. Therefore, this electro-responsive blended hydrogel patch could be useful for smart drug delivery applications.

## 1. Introduction

The transdermal drug delivery system (TDDS) is one of the most important molecule delivery systems for tissue engineering applications [1,2,3]. A drug released by TDDS can be absorbed by local peripheral tissue or bloodstream via diffusion [4]. Compared to other treatment systems, such as oral administration [5], TDDS has several advantages, such as painless administration to patients [6,7], long-term sustained release [8,9], and minimized side effects [10]. However, the inability to control drug release can lead to a risk of skin irritation and low treatment efficacy, causing the damage of normal tissues [11,12,13]. This limitation of TDDS can be addressed with the development of new drug delivery carriers, such as liposomes [14], nanoparticles [15], polymers [16], and hydrogels [17].

Hydrogels have widely been used in biomedical applications (e.g., drug delivery, wound healing, and tissue regeneration) due to their excellent properties (e.g., three-dimensional (3D) matrix structure, biocompatibility, and biodegradability) [18,19,20,21,22]. In particular, hydrogels show a number of pharmacoeconomic advantages by enabling controlled drug release in response to external stimuli [23], enhancing treatment efficacy [24], and facilitating the effective localized drug delivery system [25]. These benefits contribute to optimized treatment outcomes, improved patient compliances, and reduced healthcare costs [26,27,28]. Among the hydrogels, alginate can easily form a gel structure through crosslinking with cations. Furthermore, it enables drug loading and release due to a 3D hydrogel network generated by its interaction with multivalent metal cations (e.g., Ca^2+^, Zn^2+^, and Cu^2+^) [29,30,31]. Despite the great potential of alginate, it has some limitations, such as poor mechanical properties and uncontrollable drug release [29,32]. To overcome these limitations, many studies have been conducted on mixing alginate with various materials [33,34,35]. For instance, alginate was mixed with photo-crosslinkable gelatin methacrylate (GelMA) hydrogels to improve its mechanical strength [34]. The GelMA-alginate (Gel-Alg) hydrogel showing interpenetrative polymer networks (IPNs) represented good mechanical properties as compared to alginate or GelMA hydrogel alone. In another study, a hydrogel composed of alendronate (ALN)-modified alginate and GelMA was employed for ALN release in response to a pH environment [35]. The hydrogels containing GelMA and alginate showed good mechanical properties and external-stimuli-responsive molecule release. However, these previous hydrogels still have limitations, such as burst drug release.

To overcome the limitation of the burst drug release, electrical stimulation (ES) can be applied to the hydrogels. Recent studies have focused on integrating conductive materials into the hydrogels [36,37,38,39]. The conductive materials (e.g., polypyrrole, poly(3,4-ethylenedioxythiophene)-poly(styrenesulfonate) (PEDOT/PSS), and polyaniline) can enhance the electrical conductivity of the hydrogels. Among them, silver (Ag) shows high conductivity, good biocompatibility, and antimicrobial properties [40]. In particular, silver nanowire (AgNW) maintains conductivity under a deformation environment and provides an electrical field which can significantly affect cellular functions [41,42]. A previous study has developed conductive hydrogels to investigate the effect of ES applications on cellular functions [41]. It showed that the neural stem cells cultured on polyethylene glycol (PEG)/AgNW/reduced graphene oxide (rGO) hydrogel nanopatterns showed ES-mediated differentiation behaviors. In another study, conductive hydrogels containing AgNW and methacrylated alginate (MA) were developed [42]. This conductive hydrogel exhibited good mechanical strength, antibacterial efficacy, and cell proliferation for the acceleration of wound healing.

**Figure 1:**
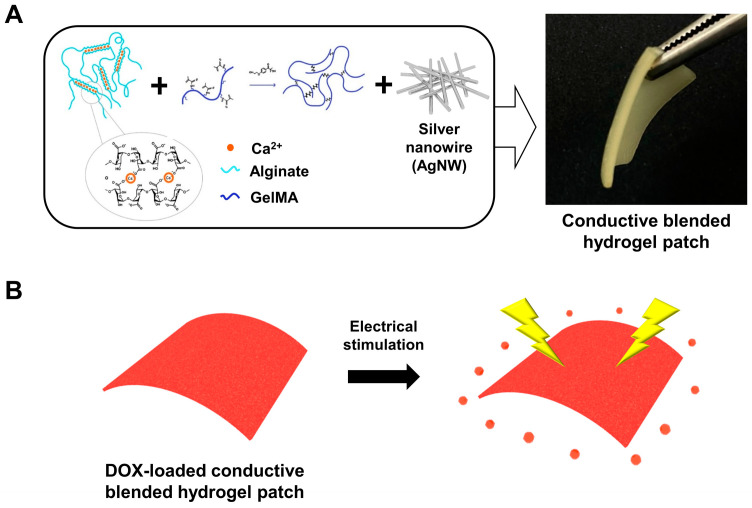
Schematic process of the electro-responsive conductive Gel-Alg-AgNW blended hydrogel and a photograph of Gel-Alg-AgNW blended hydrogel patch (**A**). Electro-responsive DOX release from conductive Gel-Alg-AgNW blended hydrogel patch (**B**).

In this study, we designed an electro-responsive conductive GelMA-alginate-AgNW (Gel-Alg-AgNW) blended hydrogel patch (Figure 1). Alginate was selected for easy drug loading and release. GelMA was blended to improve mechanical properties and AgNWs were added to enhance electrical conductivity. We confirmed that our conductive Gel-Alg-AgNW blended hydrogel patches exhibited on-off drug release and effective melanoma cell death through the release of doxorubicin (DOX) under ES application. Compared to previous hydrogels, our conductive Gel-Alg-AgNW blended hydrogel shows a number of advantages, such as ES-mediated drug release and moderate mechanical strength, indicating that our Gel-Alg-AgNW blended hydrogel patches can be a potential tool for wearable skin patch applications.

## 2. Materials and Methods

### 2.1. Preparation of Gel-Alg-AgNW Blended Hydrogel

The alginate was prepared by adding sodium alginate solution (2 wt%, Ducksan Pharmaceutical Co. Ltd., Seoul, Republic of Korea) dropwise to Calcium chloride (CaCl_2_) solution (5 wt%, Samchun Pure Chemical Co. Ltd., Gyeonggi, Republic of Korea) for 1 min. The GelMA hydrogel was prepared through a photo-crosslinking process using ultraviolet (UV) light. The GelMA precursor solution (3D materials, Busan, Republic of Korea) was exposed to UV light for 20 s using UV curing system (Excelitas, Waltham, MA, USA) to gelation and formed the hydrogel. To fabricate Gel-Alg hydrogel, the GelMA precursor solution was mixed with alginate precursor solution for homogenous dispersion using a vortex mixer (IKA, Staufen, Germany). After exposing the mixture solution to UV light for 20 s, the photo-crosslinked hydrogel was further ion-crosslinked by CaCl_2_ solution for 1 min. Finally, AgNW solution (conductive ink, Republic of Korea) was added to enhance the electrical conductivity of the Gel-Alg mixed solution. The Gel-Alg-AgNW mixture solution was exposed to UV light for 20 s and was subsequently placed in CaCl_2_ solution for 1 min for photo- and ion-crosslinking in the previous way. The crosslinked samples were washed with deionized (DI) water three times and wiped off.

### 2.2. Characterization of Gel-Alg-AgNW Blended Hydrogel

The morphology of GelMA, alginate, AgNW, and Gel-Alg-AgNW blended hydrogel was observed by scanning electron microscopy (SEM, JSM-7100f, JEOL, Tokyo, Japan). Each sample was placed in the refrigerator at 4 °C for 2 h and dried with a freeze dryer (FDU-1200, Sunil eyela Inc., Seongnam-si, Republic of Korea) at −50 °C for 24 h. The prepared samples were coated with platinum (Pt) for 5 min to suppress the charge-up phenomenon during the measurements. The elemental composition and content of Gel-Alg-AgNW blended hydrogels were measured by energy X-ray dispersive spectroscopy (EDS).

### 2.3. Mechanical and Electrical Properties of Gel-Alg-AgNW Blended Hydrogel

To evaluate the durability of the hydrogels, the tensile strength of hydrogels was measured. The tensile strength of the GelMA, alginate, Gel-Alg, and Gel-Alg-AgNW blended hydrogels prepared in a rectangular shape of 2 cm × 2 cm × 0.5 cm in size was measured by tensile strength instruments. The swelling properties of the hydrogels were investigated by gravimetric analysis. The prepared hydrogel samples were immersed in 10 mL of deionized (DI) water in a petri dish at room temperature. The immersed hydrogels were carefully taken off the petri dish at specified time intervals. The residual surface moisture on the hydrogels was carefully wiped off, showing the accuracy and reliability of subsequent measurements. The weight of the hydrogels was recorded using an electrical balance. The swelling ratio of the hydrogel was calculated by the following formula:

$$\mathrm{Swelling}\;\mathrm{ratio}\;\left(\%\right)=\frac{W_{f}-W_{i}}{W_{i}}\times 100\%$$

*W_f_* is the weight of the swollen hydrogel at the time and *W_i_* is the initial weight of the hydrogel.

To analyze the electrical properties of the hydrogels, the conductivity of hydrogels was measured. The alginate, GelMA, Gel-Alg, and Gel-Alg-AgNW blended hydrogels prepared in a rectangular shape of 2 cm × 2 cm × 0.5 cm in size were fixed on a Cr-Au patterned glass and two Pt electrodes (CHI Instruments Inc., Bee Cave, TX, USA) were plugged into the hydrogel. The resistance was measured with the CHI 660E electrochemical workstation (CHI Instruments Inc., Bee Cave, TX, USA). Electrical conductivity (σ) was calculated using the following equation [43]:

$$\sigma =\frac{1}{R}\times \frac{L}{A}$$

where σ is the electrical resistivity, *R* is the resistance of hydrogel, *A* is the cross-sectional area, and *L* is the thickness of hydrogel.

### 2.4. Electro-Responsive Drug Release of Gel-Alg-AgNW Blended Hydrogel Patch

To confirm the electro-responsive drug release of the Gel-Alg-AgNW blended hydrogel patch, the Gel-Alg-AgNW blended hydrogel patch was immersed in drug solution. Briefly, 10 mg of doxorubicin (DOX, TCI, Tokyo, Japan) was dissolved in 10 mL of phosphate buffered saline (PBS, Thermo Fisher Scientific, Waltham, MA, USA) to prepare the 1 mg/mL drug solution. The Gel-Alg-AgNW blended hydrogel patch was freeze-dried for 24 h, placed in the drug solution, and immersed for 24 h. A petri dish (Thermo Fisher Scientific, Waltham, MA, USA) containing the buffer solution (pH 5.8) and hydrogel patch was placed into a hot plate at 37 °C. To evaluate the electro-responsive drug release behavior of the hydrogel, we utilized two methods: (1) continuous voltage application and (2) on-off pulsed voltage application, applying 3 V using a function generator (AFG1062, Tektronix, Beaverton, OR, USA) to evaluate the electrical-stimuli-responsive drug release. After ES application, 2 mL of the buffer solution was taken out and 2 mL of the fresh buffer solution was added every period. The absorbance of the collected buffer solution was measured at 490 nm with UV-visible spectroscopy (UV 1800, Shimadzu, Japan).

### 2.5. Biocompatibility of Gel-Alg-AgNW Blended Hydrogel Patch

To confirm the biocompatibility of hydrogels, NIH-3T3 fibroblast cells and B16F10 melanoma cells were selected and cultured with DMEM cell culture media (Dulbecco’s Modified Eagle Medium, Thermo Fisher Scientific, Waltham, MA, USA) in a cell culture dish (Thermo Fisher Scientific, Waltham, MA, USA). A total of 1 × 10^4^ fibroblast cells and melanoma cells were seeded in 24 microwell plates (Thermo Fisher Scientific, Waltham, MA, USA) and were then incubated for 1 day. The Gel-Alg-AgNW blended hydrogel patch immersed in cell culture medium was placed on the microwell and cultured for 3 days. The qualitative viability analysis of NIH-3T3 fibroblast cells and B16F10 melanoma cells was evaluated with a live/dead assay kit (Thermo Fisher, Waltham, MA, USA). The hydrogel patch was removed from each well and the cells were washed with PBS. Afterward, the fresh medium with live/dead assay kit solution was applied and the cells were incubated for 30 min. The fluorescence images of live/dead cells were obtained with a fluorescence microscope (Olympus, Tokyo, Japan) and were subsequently analyzed by Image J (National Institute of Health, Bethesda, MD, USA) software. MTT analysis was also performed for quantitative cell viability analysis. The fresh medium with MTT agent was added to the cells. After 4 h, the medium was carefully removed and 100 µL of dimethyl sulfoxide (DMSO) was added to each well to dissolve the internalized purple formazan crystals. The absorption was measured at 595 nm using an iMark™ microplate reader (Bio-rad, Hercules, CA, USA). Cell viability was calculated using the following equation:

$$\mathrm{Cell}\;\mathrm{viability}\;\left(\%\right)=\frac{OD_{hydrogel}}{OD_{control}}\times 100\%$$

where *OD_hydrogel_* is the absorbance of the cells cultured with hydrogel, and *OD_control_* is the absorbance of cells cultured in the pure medium.

### 2.6. Drug Release of Gel-Alg-AgNW Blended Hydrogel Patch

To evaluate the cell death by drug release under ES application of the blended hydrogel patch, 2.5 × 10^4^ B16F10 cells were seeded in 24 microwell plates and were then incubated for 1 day. The freeze-dried Gel-Alg-AgNW blended hydrogel patch was sterilized by irradiating UV rays for 30 min. Then, the hydrogel patch was placed in 10 mL DMEM medium and DOX-loaded DMEM medium, respectively. The Gel-Alg-AgNW blended hydrogel patch and DOX-loaded Gel-Alg-AgNW blended hydrogel patch were placed on each microwell. A 3 V voltage was applied to the Gel-Alg-AgNW blended hydrogel patch for 10 min to observe the viability of melanoma cells at the ES condition. ES was also applied to the DOX-loaded Gel-Alg-AgNW blended hydrogel patch to confirm the release of DOX. The hydrogel patch was removed after ES application and the microwells were placed in a 5% CO_2_ incubator at 37 °C for 8 h for cellular uptake. To evaluate cell viability, the live/dead assay and MTT assay were performed in the previous way.

### 2.7. Statistical Analysis

Statistical analysis was performed using an unpaired two-tailed t-test. The *p*-values were analyzed using one-way analysis of variance (ANOVA) [44]. Differences of the tensile strength and conductivity analysis were considered statistically significant (* *p* < 0.05, ** *p* < 0.01). Additionally, the differences of the cell viability experiment were considered statistically significant (** *p* < 0.01, *** *p* < 0.001) [45].

## 3. Results and Discussion

### 3.1. Characterization of Gel-Alg-AgNW Blended Hydrogel

**Figure 2:**
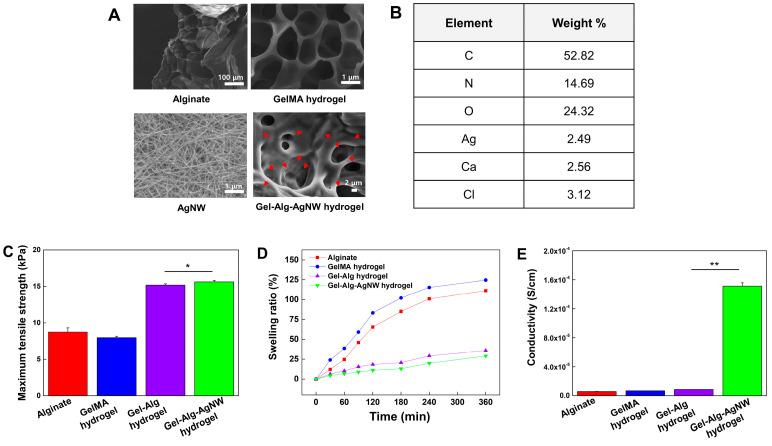
Characterizations of conductive Gel-Alg-AgNW blended hydrogel. SEM images of alginate, GelMA, AgNW, and Gel-Alg-AgNW blended hydrogel (**A**). The red arrows indicate AgNWs in the hydrogel. EDS analysis of the conductive Gel-Alg-AgNW blended hydrogel (**B**). Tensile strength (**C**), swelling ratio (**D**), and electrical conductivity (**E**) of alginate, GelMA, Gel-Alg, Gel-Alg-AgNW blended hydrogel (* *p* < 0.05, ** *p* < 0.01).

To fabricate the Gel-Alg-AgNW blended hydrogel (Figure 1), the mixing ratio was changed to determine whether the mixed hydrogel was gelated. The GelMA hydrogel precursor solution and alginate precursor solution were mixed at the ratio of Gel-Alg-AgNW of 2:8:1, 4:6:1, 5:5:1, and 6:4:1 (Figure S1). The hydrogel was not gelated when the percentage of alginate was increased. When the ratio of GelMA hydrogels was increased, the curing was stably generated due to photo-crosslinkable GelMA. Therefore, we optimized the ratio of the Gel-Alg-AgNW blended hydrogel to 6:4:1. We finally fabricated the blended hydrogels via the photo-crosslinking and ion-crosslinking processes. The morphology of the hydrogel was examined by SEM to identify the hydrogel’s structure (Figure 2A). SEM images confirmed the porous structure that could be useful for drug loading within the hydrogel. A large amount of AgNWs was also randomly distributed in the Gel-Alg-AgNW hydrogel, suggesting that the conductive network was formed by the addition of AgNWs. Furthermore, the element analysis confirmed the presence of all components required for synthesizing the Gel-Alg-AgNW blended hydrogel as shown in Figure 2B. Basic elements of hydrogels (e.g., oxygen, carbon, and nitrogen) and crosslinking agents (e.g., calcium and chlorine) were observed. Moreover, it was observed that Ag was present in the Gel-Alg-AgNW blended hydrogels, confirming that our Gel-Alg-AgNW blended hydrogels could be used as an electro-responsive polymeric material.

To evaluate the durability of our blended hydrogels, we generated a thin Gel-Alg-AgNW blended hydrogel patch (Figure 1A) and analyzed its tensile strength (Figure 2C). We observed that the Gel-Alg hydrogel showed higher tensile strength than alginate or GelMA hydrogel due to the formation of IPN combining the polymer chains of alginate and GelMA, as was previously described [34,46]. Additionally, the tensile strength of Gel-Alg-AgNW blended hydrogels was slightly increased as compared to other hydrogels due to the strength of the hydrogel network by the addition of AgNW [40,47].

We also analyzed the swelling ratio of the blended hydrogel patch (Figure 2D). The swelling of the hydrogel is a crucial factor for analysis of drug loading and molecule diffusion [48,49]. However, the excessive swelling of the hydrogel patch affects the structure and shape of the hydrogel, causing poor adhesion and inconsistent drug release [47,50]. Therefore, the swelling ratio needs to be optimized for efficient and controlled drug release. GelMA hydrogels showed the highest swelling ratio (124%), while alginate was expanded by 111% after 6 h. In contrast, the swelling ratio of Gel-Alg hydrogels was 36% due to formation of crosslinked hydrogel networks [51,52], indicating that Gel-Alg hydrogels showed excellent stability in the aqueous environment. Moreover, the addition of AgNW to the Gel-Alg hydrogel slightly reduced the swelling ratio to 29%, indicating that AgNW increased the crosslinking density of the hydrogel and alleviated the swelling [47].

To evaluate the electrical properties of the hydrogels, the conductivity of alginate, GelMA, Gel-Alg and Gel-Alg-AgNW blended hydrogels was investigated (Figure 2E). The conductivities of alginate, GelMA, and Gel-Alg hydrogels were measured to be 5.79 × 10^6^ S·m^−1^, 6.73 × 10^6^ S·m^−1^, and 8.56 × 10^6^ S·m^−1^, respectively. These results suggest that the combination of GelMA and alginate does not affect the conductivity, as previously described [53,54]. In contrast, the addition of AgNW to Gel-Alg hydrogels resulted in an 18-fold increase in conductivity (1.51 × 10^4^ S·m^−1^). Previous studies have shown that incorporating AgNWs into hydrogels improved mechanical properties through high crosslinking density and enhanced electrical properties through the formation of conductive networks. However, the crosslinking density and swelling ratio are inversely proportional [55], resulting in slow drug release [56]. Therefore, we designed Gel-Alg-AgNW blended hydrogels with high electrical conductivity and moderate mechanical strength.

### 3.2. Electrical Stimuli-Responsive Drug Release of Gel-Alg-AgNW Blended Hydrogel Patch

**Figure 3:**
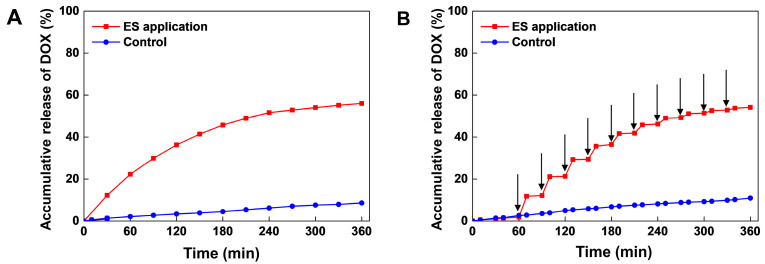
Electro-responsive drug release of DOX-loaded Gel-Alg-AgNW blended hydrogel patch. Drug release study of DOX in continuous ES application (**A**). Drug release study of DOX in on-off ES application (**B**). The arrows (↓) indicate the application of an electric potential of 3 V for 10 min, performing every 30 min.

To evaluate electro-responsive drug release behavior of the blended hydrogel patch, we performed ES experiments with continuous voltage and on-off pulsed voltage. As shown in Figure 3A, the Gel-Alg-AgNW blended hydrogel patch released only a small amount of DOX (11%) in the absence of ES application. However, cumulative release significantly increased to 60% when the continuous voltage was applied to the hydrogels. To further confirm the electrical responsive drug release behavior, we conducted an on-off drug release experiment (Figure 3B). A 3 V voltage was applied to the DOX-loaded blended hydrogel patch for 10 min every 30 min over 6 h. We observed that 11% DOX was released in the first cycle and was gradually decreased with each subsequent cycle. A total of 9% and 8% of DOX was released at the second and third cycle, respectively. This was probably due to the decrease in the DOX concentration difference between the hydrogel and PBS solution, as previously described [57]. The total amount of Dox by ES application reached 57% which was similar to continuous voltage application. It was confirmed that our Gel-Alg-AgNW blended hydrogel patch enabled the control of on-off drug release in response to ES application.

There are two mechanisms of drug release in the Gel-Alg-AgNW blended hydrogel patch. First, the movement of molecules can be manipulated by the electric field. In general, negatively charged drugs are released by a reduction of the drug carrier, whereas positively charged drugs are released by oxidation [57,58]. The DOX used in this experiment was a positively charged drug [59]. When the ES application was generated, the movement of free counter ions in the electric field could create a high osmotic pressure inside the hydrogel structure to release the DOX [60]. Second, the structure of the hydrogel network can be changed by the redox reaction [61,62]. As the hydrogel was swelled and collapsed in the presence of ES application, the oxidation-reduction state of the components was switched to generate the structural transformation [63,64]. The drug was slowly released in absence of ES application due to interactions between the drug and the hydrogel through hydrogen bonding or van der Waals forces [61]. In contrast, when ES was applied, the hydrogel structure could be changed and molecule release was accelerated [65]. Therefore, the electro-responsive drug release in the Gel-Alg-AgNW blended hydrogel patch was achieved through osmotic pressure and structural changes of hydrogel in response to ES application.

### 3.3. Biocompatibility and Drug Release of Gel-Alg-AgNW Blended Hydrogel

**Figure 4:**
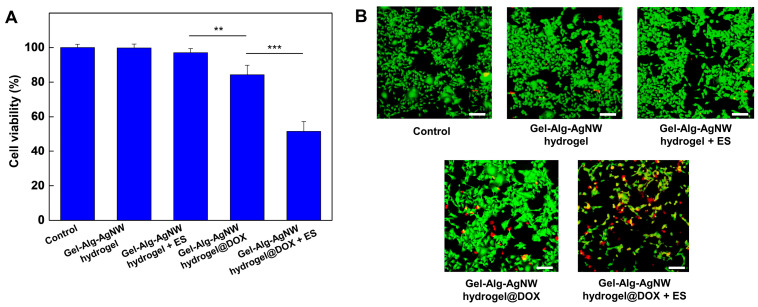
Drug release analysis of the Gel-Alg-AgNW blended hydrogel patch. Quantitative analysis of the viability of B16F10 melanoma cells in the Gel-Alg-AgNW blended hydrogel and DOX-loaded Gel-Alg-AgNW blended hydrogel patches (** *p* < 0.01, *** *p* < 0.001) (**A**). Fluorescence images of the live/dead assay in B16F10 melanoma cells on the Gel-Alg-AgNW blended hydrogel and DOX-loaded Gel-Alg-AgNW blended hydrogel patches (**B**). The live and dead cells were stained with Calcein AM (green) and Ethidium homodimer-1 (red), respectively. Scale bars are 100 μm.

To evaluate the biocompatibility of the Gel-Alg-AgNW blended hydrogel patch, NIH-3T3 fibroblast cells and B16F10 melanoma cells were selected and cultured (Figure S2). After culturing the cells for 1 day, the blend hydrogel patch was placed in a cell culture well and was further cultured for 1 day and 3 days to confirm cell viability. It showed that the cell viability of fibroblasts and melanoma cells on day 1 was above 95% regardless of the presence of the blended hydrogel patch. Furthermore, no significant difference was found in cell viability on day 3 compared to day 1. These experimental results indicate that our blended hydrogel patches have high biocompatibility. We also conducted an electrical-stimuli-responsive drug release experiment to confirm the cell death effect by drug release under ES application (Figure 4). We investigated the cell viability in the Gel-Alg-AgNW blended hydrogel patch in response to ES application. It showed that there was no significant difference in cell viability between the control and ES application, indicating that ES application did not affect cell viability. Additionally, we compared cell viability in the DOX-loaded Gel-Alg-AgNW blended hydrogel patch in response to ES application. When the DOX-loaded hydrogel patch was used in absence of ES application, the cell viability was reduced to 84% because DOX in the blended hydrogel was naturally released. In contrast, the cell viability was dramatically decreased to 51% when ES was applied to the DOX-loaded Gel-Alg-AgNW blended hydrogel patch. To observe the therapeutic effect of the DOX-loaded Gel-Alg-AgNW blended hydrogel patch on melanoma cells, the live/dead assay was performed (Figure 4B). The cell viability of melanoma cells was examined in response to ES application as a control group. We observed that most cells were live, indicating that ES application did not significantly affect cell viability. In contrast, the melanoma cells were dead in response to ES application because DOX in the Gel-Alg-AgNW blended hydrogel patch was released in a controlled manner.

## 4. Conclusions

We developed an electro-responsive Gel-Alg-AgNW blended hydrogel patch showing excellent electrical-stimuli-responsive on-off controlled drug release. The mixing ratio of Gel-Alg-AgNW blended hydrogel was optimized and SEM images and EDS analysis of the conductive blended hydrogel showed that AgNWs were randomly distributed in the blended hydrogel, suggesting that our hydrogel can be used for electro-responsive polymeric material. The tensile strength of our blended hydrogel was increased by a factor of 1.8 and its electrical conductivity was increased by a factor of 18 with the addition of GelMA and AgNW, suggesting that it showed high electrical conductivity and moderate mechanical strength. The Gel-Alg-AgNW blended hydrogel patch enabled controllable drug release in response to on-off ES application. The cell viability analysis also demonstrated that the skin melanoma cells showed low viability (51%) through electro-responsive DOX release. Therefore, this electro-responsive Gel-Alg-AgNW blended hydrogel patch could be useful for wearable skin patch applications.

## Data Availability

The data presented in this study are available upon request from the corresponding author.

## Supplementary Materials

The following are available online at https://www.mdpi.com/article/10.3390/polym15122608/s1. Figure S1: Analysis of the gelation of the hydrogel with respect to the GelMA/alginate/AgNW ratio. Figure S2: Quantitative analysis of viability of NIH-3T3 fibroblast cells (A) and B16F10 melanoma cells (B) cultured with the Gel-Alg-AgNW-blended hydrogel patch. The cell viability was evaluated on both day 1 and day 3 of the experiment.
