# Supplementary material for: Electro-Responsive Conductive Blended Hydrogel Patch
Source: Polymers (Basel). 2023 Jun 8;15(12):2608. doi: 10.3390/polym15122608 (PMC10300816; doi:10.3390/polym15122608)
Supplement: Supplementary file 1 [file polymers-15-02608-s001.zip › polymers-2395213-supplementary.pdf]

polymers

MDPI

Supporting Information

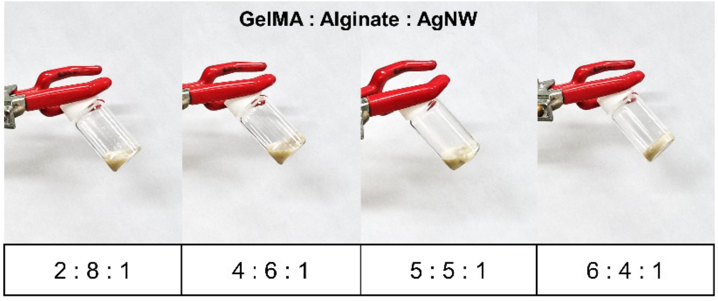

Figure S1. Analysis of the gelation of the hydrogel with respect to the GelMA/alginate/AgNW ratio

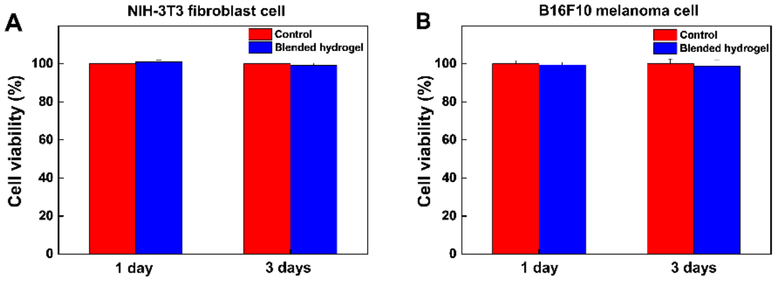

Figure S2. Quantitative analysis of viability of NIH-3T3 fibroblast cells (A) and B16F10 melanoma cells (B) cultured with the Gel-Alg-AgNW blended hydrogel patch. The cell viability was evaluated on both day 1 and day 3 on the experiment.
